# Blood pressure estimation system using human body communication-based electrocardiograph and photoplethysmography

**DOI:** 10.1049/htl.2019.0105

**Published:** 2020-06-23

**Authors:** Yusuke Sawatari, Jianqing Wang, Daisuke Anzai

**Affiliations:** Nagoya Institute of Technology, Nagoya 466-8555, Japan

**Keywords:** medical signal processing, electrocardiography, blood, blood pressure measurement, photoplethysmography, synchronisation, correlation coefficient, root mean square error, automatic synchronisation, R-wave, pulse arrive time, low-load cuffless blood pressure measurement, continuous blood pressure estimation, cuffless blood pressure estimation, measured electrocardiograph, pulse wave signals, time period, systolic blood pressure, reflectance photoplethysmography, human body communication-based wearable electrocardiograph, continuous blood pressure measurement, blood pressure estimation system

## Abstract

In order to realise low-load cuffless and continuous blood pressure measurement in daily life, the authors developed a blood pressure estimation system combining human body communication-based wearable electrocardiograph and reflectance photoplethysmography. The principle is based on a relationship between the pulse arrive time and the systolic blood pressure. The pulse arrive time is the time period between the R-wave in electrocardiograph and peak of pulse wave. The greatest feature is the use of a human body communication-based electrocardiograph which can provide automatic synchronisation in time between the measured electrocardiograph and pulse wave signals to obtain the pulse arrive time so that no additional synchronisation circuit is required. Using this system, the authors measured the pulse arrive time from the electrocardiograph and pulse wave signals in real time, estimated the systolic blood pressure and compared the result with that measured by a cuff sphygmomanometer. The authors found that the root mean square error of the estimated blood pressure and the actual value measured using the cuff sphygmomanometer was 4.5 mmHg or less, and the correlation coefficient was >0.6 with a *P* value much <0.05. These results show the validity of the developed system for cuffless and continuous blood pressure estimation.

## 1 Introduction

With the progress of the aging society, low-load and real-time vital signal monitoring technology with the help of body area networks has attracted much attention for medical and health care applications [1–3]. Some typical examples are to monitor various vital signals such as blood pressure (BP), electrocardiogram (ECG) and pulse rate during daily life or driving using wearable sensors with a wireless communication function. Hypertension is one of major problems especially for the elderly, and it is necessary to grasp the BP without stressing them. For BP measurement, the oscillometric method using pressurisation with a cuff is conventionally used [4–6]. The cuff pressure is high pass filtered to extract small oscillations at the cardiac frequency, and the envelope of these oscillations is calculated as the area obtained by integrating each pulse. As the cuff pressure drops between systolic BP and mean arterial pressure, these oscillations in the cuff pressure increase in amplitude. The amplitude of oscillations then decreases when the cuff pressure falls below mean arterial pressure. The corresponding oscillation envelop function is interpreted by computer-aided analysis to extract BP. However, there is a load on the subject and a difficulty to constantly measure the BP's fluctuation. A BP measurement method which does not require cuff pressure is more practical during daily life or driving. One of alternative BP measurement methods is based on pulse arrive time (PAT) [7–10], which is the time for a pulse of blood to travel from the heart to the organ where measurement is undertaken. This method requires at least two vital signals, one as a time reference and the other to obtain the time delay, in order to calculate the PAT, from vital sensors placed on the human body. The two signals are commonly ECG and pulse wave (PW). The PW is a change in blood vessel volume which occurs as the heart pumps blood. Photoplethysmography (PPG) is most popularly used to measure PW [10–12]. PPG usually has two modes – transmission and reflectance. In transmission mode, the light transmitted through the medium is detected by a photo diode (PD) opposite the light emitting diode (LED) source, while in reflectance mode, the PD detects light that is back-scattered or reflected from the blood vessels. So reflectance PPG is based on the principle that light reflection changes as the blood vessel volume changes. Compared to transmission PPG, reflectance PPG is more suitable for daily and wearable use because of its great freedom of sensor placement. With the measured PAT from ECG and PW, BP can be estimated based on its correlation to PAT.

In view of the necessary to form a body area network for medical and health care applications, the on-body vital sensors should have a wireless function. Since the human body is a lossy dielectric body, the on-body path loss becomes larger at higher frequencies. Compared to the popularly used 2.4 GHz band for short-range communications, human body communication (HBC) technology exhibits more advantages in not only its low propagation loss compared to 2.4 GHz band but also its high security because of low radiation towards outside of the human body [1, 13, 14]. We have previously developed a wearable ECG module based on wide band HBC technology [15, 16]. For a person wearing the HBC-based ECG module with two electrodes attached on the chest, when his/her fingertip touches a HBC receiver, the detected ECG signal will be transmitted to the receiver from the chest to the fingertip through the human body itself. At the same time, the reflectance PPG can measure the PW at the fingertip, which comes from the travel of a pulse of blood from the chest to the fingertip. If we employ an HBC-based wearable ECG module and a reflectance PPG module, the ECG and PW can be measured at the same time at the hand. This means that the HBC-based wearable ECG and reflectance PPG have good compatibility with simultaneous measurement of ECG and PW, and thus are especially suitable for estimating BP from PAT.

In this study, we aim to develop a system for cuffless BP estimation from HBC-based ECG and reflectance PPG. Although the concept of estimating BP from PAT is not new, the use of HBC-based ECG to obtain PAT is a new attempt. This study is organised as follows. Section 2 describes the principle of BP estimation from PAT. Section 3 describes the configurations of HBC-based wearable ECG module and reflectance PPG module, respectively. Section 4 gives the experimental verification results and Section 5 concludes this study.

## 2 Principle

**Fig. 1:**
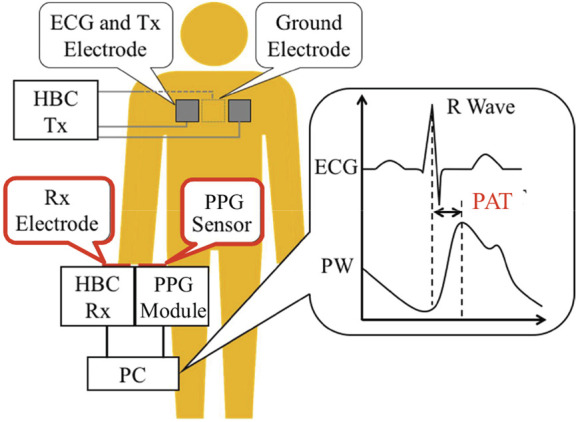
Schematic representation of BP measurement using HBC-based ECG and reflectance PPG

Fig. 1 shows a schematic representation of BP measurement from the HBC-based ECG and reflectance PPG. A pair of ECG signal and PW signal is acquired simultaneously by using the left or right hand to touch an HBC receiver electrode and a PPG sensor module. For the ECG and PW signals obtained through the hand in Fig. 1, the ECG signal is actually a time delay of that measured at the chest, which is transmitted to the hand along the human body by HBC technology. On the other hand, the PW signal is due to the blood flow reached from the heart to the hand when the heart pumps blood. The pumping timing corresponds to the R-wave in the ECG signal. So the two signals are automatically synchronised in time, and no additional synchronisation circuit is required. This attributes to the use of HBC-based ECG and is the greatest feature of the proposed system. Referring to Fig. 1, the PAT is defined as the time period between the R-wave in the ECG signal and the peak in the PW signal. Strictly speaking, it is somewhat shorter than the strict definition of PAT because the ECG signal measured by the HBC technology has a slight time delay than that measured at the chest. However, this influence can be absorbed in the coefficients of the estimation formulas by prior calibration as shown below.

According to Moens–Korteweg formula, the PAT $T_{\rm P}$ can be linked to the modulus of elasticity *E* of the blood vessel wall by [17]

(1) $$\displaystyle{{\Delta x} \over {T_{\rm p}}} = \sqrt {\displaystyle{{Eh} \over {2\rho r}}} \eqno\lpar 1\rpar $$

where $\Delta x$ is the distance between the ECG acquisition position and the PW acquisition position, *h* is the thickness of blood vessel wall, $\rho $ is the blood density and *r* is the inner radius of blood vessel. On the other hand, the systolic BP is related to the modulus of elasticity *E* of blood vessel wall by [18]

(2) $$E = E_0\exp \lpar \alpha _0P_{\rm s}\rpar \eqno\lpar 2\rpar $$

where $E_0$ and $\alpha _0$ should be determined from experimental results and $P_{\rm s}$ is the systolic BP. It should be noted that this formula applies to systolic BP, so the method presented below is not directly applicable to diastolic BP. From (1) and (2), we can derive a relationship between the systolic BP $P_{\rm s}$ and the PAT $T_{\rm p}$ as follows:

(3) $$P_{\rm s} = \alpha _1\ln T_{\rm p} + \alpha _0\eqno\lpar 3\rpar $$

where $\alpha _1$ and $\alpha _0$ are two coefficients related to *E*, *h*, $\rho $, *r* and $\Delta x$.

Moreover, it is suggested that there is a linear relationship between the BP and heart rate. Since we can obtain the heart rate from the acquired ECG signal in real time, also taking this factor into account is expected to improve the estimation accuracy. We thus add a new term related to the heart rate in (3), i.e.

(4) $$P_{\rm s} = \alpha _1\ln T_{\rm P} + \alpha _2T_{\rm h} + \alpha _0\eqno\lpar 4\rpar $$

where $T_{\rm h}$ is the cardiac cycle in unit of second.

Therefore, if the coefficients $\alpha _1$, $\alpha _2$ and $\alpha _0$ are derived by prior calibration, the systolic BP can be estimated from PAT using (3) or (4).

## 3 System configuration

### 3.1 HBC-based wearable ECG module

**Fig. 2:**
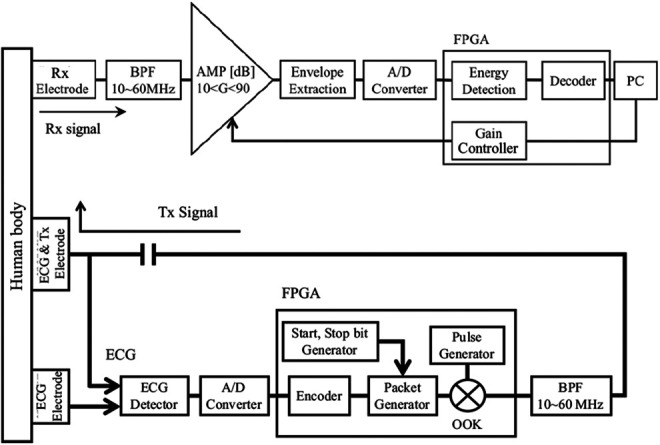
Block diagram of the HBC-based wearable ECG module

Fig. 2 shows the block diagram of the proposed HBC-based wearable ECG module. First, the ECG signal is acquired by a pair of electrodes and a detection circuit, and then is converted to a digital signal by an analogue-to-digital (AD) converter. Next, each digital bit is encoded by a known pseudo noise sequence consisting of eight chips, which is actually an eight-fold direct spreading code. The encoded data are formed to a packet with a definite length by adding start and stop bits before and after them, respectively. The modulation employs impulse radio (IR) scheme where a pulse with a width of 100 ns is used to replace usual carrier signal. The pulse is sent when the chip is ‘1’, and nothing is sent when the chip is ‘0’, which is actually an encoded on–off keying (OOK) modulation. The chip rate is 10 Mc/s, which corresponds to a bit rate of 1.25 Mb/s. At the output of the transmitting part, the modulated signals are spectrally shaped between 10 and 60 MHz by a band pass filter (BPF). The signal to be transmitted (Tx signal) is then added to the human body by the same electrodes for ECG detection, and is transmitted to the receiving part through the human body. After BPF and amplification, the received signals are demodulated by an energy detector and decoded to extract the original bits. By employing the wide band signal of 10–60 MHz, the usual 2.4 GHz band is avoided and high anti-electromagnetic interference performance can be expected. In fact, for our developed HBC-based wearable ECG module, the electric field strength was measured not to exceed 25.4 V/m at a distance of 3 m, smaller than usual electromagnetic environment [19]. This suggests that the electromagnetic radiation and information leakage to the outside of the human body are very weak because of the use of human body as a transmission route.

Table 1 summarises the specifications of the HBC-based wearable ECG module. Both the transmitting and receiving parts are implemented in a field-programmable gate array, respectively. The sizes of the wearable part are about 3 cm × 3 cm. It is stored in a plastic case. The consumption power is <5 mW, and the weight is <10 g. The ECG module starts operating when the power is turned on, and no warm-up time is required. After charging, it can work for at least half a day, or 4 h. A typical ECG measurement location, i.e. the Tx location, is on the chest and is particularly recommended for extended use due to the stability of the electrodes when in contact with human skin. The communication distance from the Tx location on the chest to the finger (Rx location) therefore does not change greatly so that the consumption power almost does not depend on the ECG measurement location. We made continuous measurements for up to four hours, and no significant increase in temperature was observed.

**Table 1:** Specifications of HBC-based wearable ECG module

ECG detector	
HPF cutoff frequency	15.9 Hz
LPF cutoff frequency	88.4 Hz
amplification	60 dB
AD converter	—
sampling frequency	500 Hz
quantisation level	10 bits
Tx	—
frequency band	10–60 MHz
pulse width	100 ns
chip rate	10 Mc/s
chip number per bit	8
bit rate	1.25 Mb/s
modulation	IR OOK
Rx	—
amplification	60 dB automatic gain control
demodulation	energy detection
output interface	USB

### 3.2 Reflectance PPG module

**Fig. 3:**
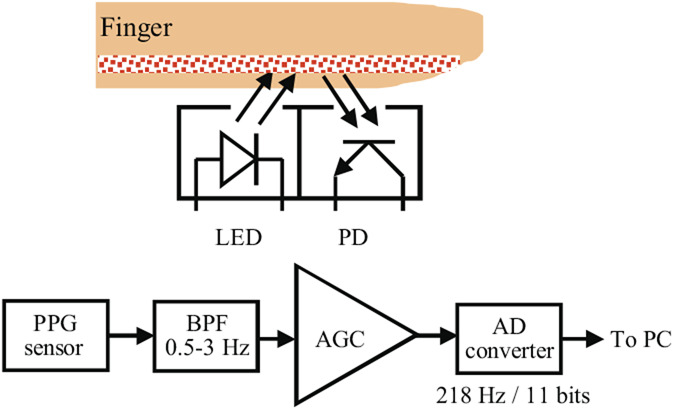
Block diagram of the reflectance PPG module

Reflectance PPG is based on optical detection of blood volume changes in the micro-vascular bed of the tissue. A reflectance PPG sensor consists of an infrared LED as the light source and a PD as the light detector. They are placed in proximity to each other on the skin surface where there is a detectable concentration of blood vessels. The sensor monitors changes in the light intensity via reflection from the tissue. The changes in light intensity are associated with small variations in blood perfusion of the tissue and provide information on the pulse rate. Fig. 3 shows a block diagram of the reflectance PPG module. The PW is detected by pressing the finger on the sensing part. The detected PW is passed through a BPF of 0.5–3 Hz and amplified to an appropriate level by an amplifier with an automatic gain control function. The peaks of the PW waveform are usually not very sharp. Typically, a PW waveform has two peaks. One is produced by the draining of blood when the heart contracts and the other is a reflected wave in which the PW propagates to the fingertip and reflects back. The former peak is usually larger than the latter. In order to effectively extract the former peak of the PW waveform, the reflective PPG sensor has a differentiator circuit in which the normal PW waveform is differentiated and then amplified. This is called acceleration PW which makes the inflection points of the PW clearer so that the former peak can be effectively emphasised. After that, the PW is quantised by 11 bits in the range of 0–5 V by an AD converter with a sampling frequency of 218 Hz, and sent to a personal computer (PC) via the USB cable. On PC, the peak of the acceleration PW is identified using a usual peak search algorithm. Since reflectance PPG is based on the principle that light reflection changes as the blood vessel volume changes, the finger's multilayer configuration is included in the prior calibration process even if there are differences in the multilayer configuration for different subjects. It is therefore possible to uniquely determine the relationship between BP and PAT.

## 4 Verification experiment

Using the developed HBC-based wearable ECG module and the reflectance PPG module, we experimentally verified the validity of systolic BP estimation from the simultaneously measured ECG and PW waveforms. The simultaneous measurement of ECG and PW was realised by touching the HBC-based ECG receiving electrode with a hand and the reflectance PPG sensor with one of its fingers at the same time. Then the PAT *T*_P_ was obtained, and the systolic BP was estimated using (3) or (4). The estimated systolic BP values were compared with that measured using a cuff sphygmomanometer to verify the method's validity.

**Fig. 4:**
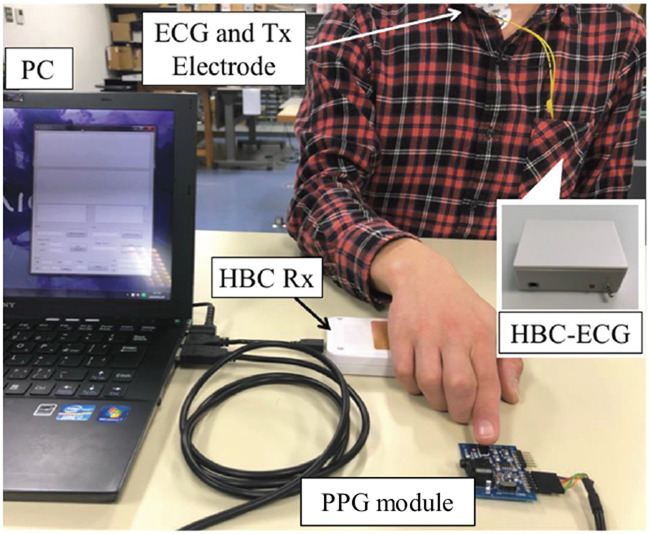
View of simultaneous measurement of ECG and PW

**Fig. 5:**
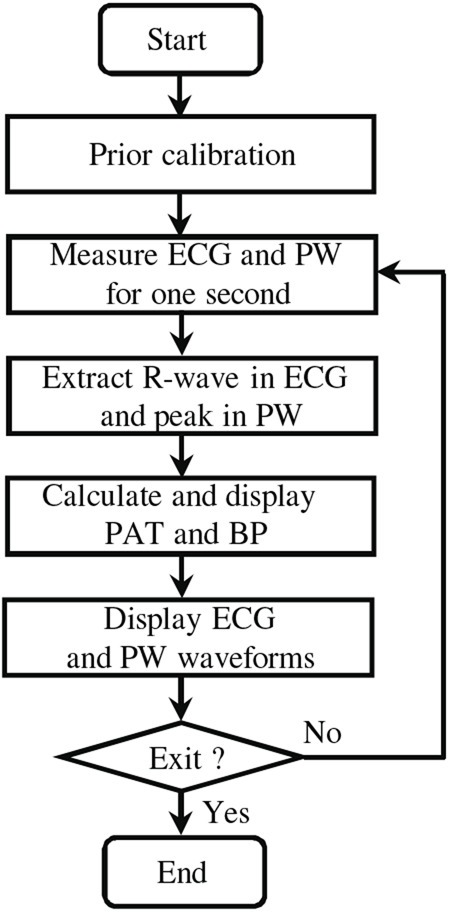
Flow chart of BP estimation

**Fig. 6:**
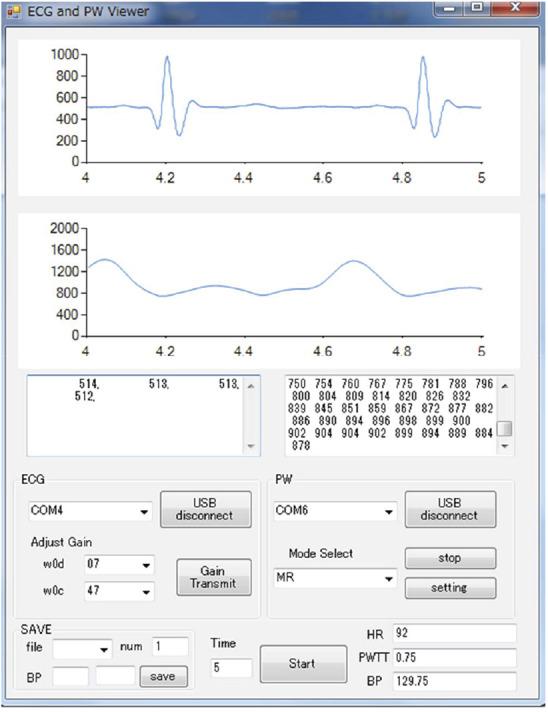
Example of screen showing measured ECG and PW waveforms

Fig. 4 shows a view of simultaneous measurement of ECG and PW waveforms. The HBC-based wearable ECG is attached on the subject's chest with the electrodes touching the chest surface. During measurement, the subject's left or right hand touches the receiving electrodes of the HBC-based wearable ECG, and one finger simultaneously touches the PPG sensor. There is no clear difference between using left hand and right hand in terms of performance. Fig. 5 shows the flow chart of systolic BP estimation. Before measurement, we need to conduct a prior calibration in order to determine the coefficients in (3) and (4). After the calibration, we start the measurement. First, we acquire the ECG and PW waveforms for about one second. Then, we extract the R-wave in ECG waveform and the peak in PW waveform, and calculate the time between the two peaks. This time is the PAT. Using the obtained PAT, we estimate the systolic BP from (3) or (4). This process is conducted in a PC. The measured ECG and PW waveforms, as well as the calculated PAT and estimated systolic BP are displayed on the PC's screen. Fig. 6 shows an example of screen with the simultaneously measured ECG and PW waveforms as well as the estimated BP. As can be seen, the ECG and PW waveforms are well measured, and the PAT can be easily detected from the R-wave in ECG and the peak of the PW waveform. In addition, in view of the high data rate in our HBC-based wearable ECG and the simultaneous acquisition of the ECG and PW waveforms, we do not require additional circuit to synchronise the two waveforms in order to obtain the PAT.

### 4.1 Prior calibration

**Fig. 7:**
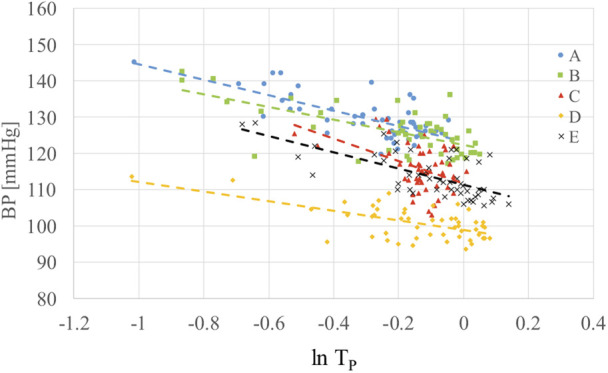
Relationship between the systolic BP and the PAT for five subjects

The verification experiment was conducted for five subjects. The five subjects were men aged 21–24 years old, except for one with mild hypertension in 50 s. For prior calibration, we measured the ECG and PW for >30 times (more than one second each time), and calculated the PAT and systolic BP for each subject. On the other hand, we obtained the systolic BP from the average before and after each ECG and PW measurement using a cuff sphygmomanometer. Fig. 7 shows the relationship between $\ln T_{\rm P}$ and systolic BP $P_{\rm s}$ based on multiple measurements for the five subjects (A–E). Since there is an approximate linear relationship between $\ln T_{\rm p}$ and systolic BP $P_{\rm s}$, we made a regression analysis to determine the coefficients in the equations. Table 2 shows the determined coefficients $\alpha _1$, $\alpha _2$ and $\alpha _0$ in (3) and (4) for BP estimation for the five subjects. Although the coefficients are different for each subject, the differences do not exceed 2.4 times for $\alpha _1$ and 1.3 times for $\alpha _0$ at maximum. For the term associated with $\alpha _2$ in (4), it has a smaller contribution to the BP estimation result compared to the terms associated with $\alpha _1$ and $\alpha _0$.

**Table 2:** Coefficients determined in (3) and (4) for BP estimation

Subject	*A*	*B*	*C*	*D*	*E*
$\alpha _1$	−21.31	−17.49	−30.66	−13.16	−22.49
$\alpha _0$	123.30	122.35	111.76	98.87	111.36
$\alpha _1$	−21.54	−16.92	−32.68	−13.51	−18.50
$\alpha _2$	13.75	−6.99	8.76	1.60	−17.53
$\alpha _0$	112.36	128.80	104.13	97.39	127.38

### 4.2 Verification results

**Fig. 8:**
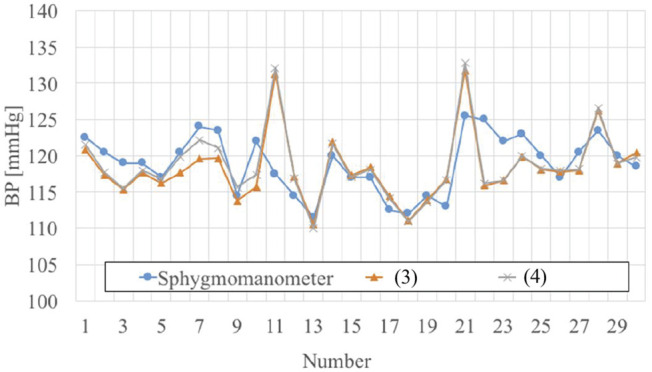
Comparison between the systolic BP estimated from PAT and that measured by a cuff sphygmomanometer. The numbers on the horizontal axis indicate the measurement number

The BP estimation was made using either (3) or (4). That is to say, we first obtained instantaneous PAT value, and then used them to estimate BP from either (3) or (4). The reference values of BP were an average measured by a cuff sphygmomanometer before and after the ECG and PW measurement. Fig. 8 compares the systolic BPs estimated from PAT and measured by cuff sphygmomanometer for one subject. The test was conducted 30 times in total within three days. The time duration of each test was 30 seconds. In Fig. 8, the numbers on the horizontal axis indicate the test number, and the vertical axis indicates the systolic BP value averaged within 30 s. It can be seen that the total variations of BP values estimated from either (3) or (4) follow the trend measured by the cuff sphygmomanometer. In both cases of using (3) and (4), the root mean square error (RMSE) was calculated between the estimated and the measured BP values, and the calculated RMSE results are shown in Table 3. The RMSE was found to not exceed 4.52 mmHg, and the systolic BP was estimated in a reasonable accuracy because there is no significant difference in the estimated BP and the cuff-sphygmomanometer-measured BP.

**Table 3:** RMSE [mmHg] for estimated and measured BPs

Eq.	*A*	*B*	*C*	*D*	*E*
(3)	2.68	4.52	4.15	2.91	2.12
(4)	2.57	4.43	4.03	2.87	1.97

Moreover, the correlation coefficients were also calculated for estimated and measured BP values along with a *P* value based on a *t*-test. *P*-value is a reference probability to determine whether or not a null hypothesis (no correlation between them) can be rejected, and that the *P*-value of 0.05 is usually used as a significance level for a statistical test. First, it is assumed that there is no correlation between the estimated and measured BP values. Then, if $P \lt 0.05$, the null hypothesis will be rejected, and a significant correlation should exist between them. Table 4 shows the correlation coefficients and *P* values for the estimated BPs and that measured by the cuff sphygmomanometer. The correlation coefficients were found to be >0.55 using (3) and 0.6 using (4) for all subjects. Also, the *P*-value was always much <0.05, which means that the correlations are sufficiently significant from a statistical point of view. In addition, it was also confirmed that the correlation coefficient can be improved if the BP is estimated using (4) in consideration of heart rate as compared with (3).

**Table 4:** Correlation coefficients and *P* values

Eq.	*A*	*B*	*C*	*D*	*E*
(3)	0.55	0.70	0.55	0.65	0.57
—	$1.6 \times 10^{ - 3}$	$1.9 \times 10^{ - 5}$	$1.7 \times 10^{ - 3}$	$8.8 \times 10^{ - 5}$	$9.7 \times 10^{ - 4}$
(4)	0.65	0.71	0.60	0.66	0.60
—	$1.1 \times 10^{ - 4}$	$9.9 \times 10^{ - 6}$	$5.0 \times 10^{ - 4}$	$6.4 \times 10^{ - 5}$	$7.1 \times 10^{ - 4}$

upper row: correlation coefficient; lower row: *P*-value.

## 5 Conclusion

In this study, we have developed a systolic BP estimation system which combines an HBC-based wearable ECG module and a reflectance PPG module to realise a low-load cuffless BP measurement in daily life. The developed HBC-based wearable ECG automatically synchronises with PW because it uses the human body itself as a transmission route. This is the greatest feature of this system and no additional synchronisation circuit is required. Furthermore, by adopting a wide band IR modulation method between 10 and 60 MHz, the anti-electromagnetic interference performance has also been improved. Using the developed system, we simultaneously measured the ECG and PW waveforms, estimated the systolic BP form derived PAT, and compared the result with that measured by a cuff sphygmomanometer. We have found that the RMSE of the estimated BP and the actual measured one was 4.5 mmHg or less, and the correlation coefficient was >0.6 with a statistical *P*-value much <0.05. These results show the validity of the developed system for cuffless and continuous BP estimation.

This method is expected to be contactless. However, the capacitive-coupling impedance between the ECG electrodes and the human body can have a significant impact on signal detection quality [20], and both noise cancellation and body movement removal techniques are required [21, 22]. The integrated circuit design of HBC-based ECG and reflectance PPG modules using these techniques is our future subject.

## References

[1] Wang J. Wang Q.: ‘Body area communications’ (Wiley-IEEE, Singapore, 2012)

[2] Bonato P.: ‘Wearable sensors and systems - from enabling technology to clinical applications’, IEEE Eng. Med. Biol. Mag., 2010, 29, (3), pp. 25–36 (doi: 10.1109/MEMB.2010.936554) 20659855 10.1109/MEMB.2010.936554

[3] IEEE Std 802.15.6-2012: IEEE Standard for local and metropolitan area networks - Part 15.6: Wireless Body Area Networks, 2012

[4] Geddes L.A. Baker L.E.: ‘Principles of applied biomedical instrumentation’ (Wiley, USA, 1991, 3rd Edn.)

[5] Babbs C.F.: ‘Oscillometric measurement of systolic and diastolic blood pressures validated in a physiologic mathematical model’, Biomed. Eng. Online, 2012, 11, (56) 10.1186/1475-925X-11-56 PMC3541069 22913792

[6] Soueidan K. Chen S. Dajani H.R.: ‘Augmented blood pressure measurement through the noninvasive estimation of physiological arterial pressure variability’, Physiol. Meas., 2012, 33, pp. 881–899 (doi: 10.1088/0967-3334/33/6/881) 22551623 10.1088/0967-3334/33/6/881

[7] Chen W. Kobayashi T. Ichikawa S.: ‘Continuous estimation of systolic blood pressure using the pulse arrival time and intermittent calibration’, Med. Biol. Eng. Comput., 2000, 38, pp. 569–574 (doi: 10.1007/BF02345755) 11094816 10.1007/BF02345755

[8] Buxi D. Redout J.-M. Yuce M.R.: ‘A survey on signals and systems in ambulatory blood pressure monitoring using pulse transit time’, Physiol. Meas., 2015, 36, (3), p. R1 (doi: 10.1088/0967-3334/36/3/R1) 25694235 10.1088/0967-3334/36/3/R1

[9] Mukkamala R. Hahn J. Inan O.T.: ‘Toward ubiquitous blood pressure monitoring via pulse transit time: theory and practice’, IEEE Trans. Biomed. Eng., 2015, 62, (8), pp. 1879–1901 (doi: 10.1109/TBME.2015.2441951) 26057530 10.1109/TBME.2015.2441951 PMC4515215

[10] Ding X. Yan B.P. Zhang Y.-T.: ‘Pulse transit time based continuous cuffless blood pressure estimation: A new extension and a comprehensive evaluation’, Sci. Rep., 2017, 7, (1), pp. 11554–11564 (doi: 10.1038/s41598-017-11507-3) 28912525 10.1038/s41598-017-11507-3 PMC5599606

[11] Tamura T. Maeda Y. Sekine M.: ‘Wearable photoplethysmographic sensors: past and present’, Electronics, 2014, 3, (2), pp. 282–302 (doi: 10.3390/electronics3020282)

[12] Lee Y. Shin H. Jo J.: ‘Development of a wristwatch-type PPG array sensor module’. Proc. IEEE Int. Conf. on Consumer Electronics, Berlin, Germany, 6–8 September 2011, pp. 168–171

[13] Baldus H. Corroy S. Fazzi A.: ‘Human-centric connectivity enabled by body - coupled communications’, IEEE Commun. Mag., 2009, 47, (6), pp. 172–178 (doi: 10.1109/MCOM.2009.5116816)

[14] Wang J. Nishikawa Y. Shibata T.: ‘Analysis of on-body transmission mechanism and characteristic based on an electromagnetic field approach’, IEEE Trans. Microw. Theory Tech., 2009, 57, (10), pp. 2464–2470 (doi: 10.1109/TMTT.2009.2029632)

[15] Wang J. Fujiwara T. Kato T.: ‘Wearable ECG based on impulse radio type human body communication’, IEEE Trans. Biomed. Eng., 2016, 63, (9), pp. 1887–1894 (doi: 10.1109/TBME.2015.2504998) 26642315 10.1109/TBME.2015.2504998

[16] Ando H. Murase Y. Anzai D.: ‘Wireless control of robotic artificial hand using myoelectric signal based on wideband human body communication’, IEEE Access, 2019, 7, pp. 10254–10262 (doi: 10.1109/ACCESS.2019.2891723)

[17] Bramwell J.C. Hill A.V.: ‘The velocity of the pulse wave in man’, Proc. Royal Soc. London B, Biol. Sci., 1922, 93, (652), pp. 298–306

[18] Hughes D.J. Babbs C.F. Geddes L.A.: ‘Measurements of young's modulus of elasticity of the canine aorta with ultrasound’, Ultrason. Imag., 1979, 1, (4), pp. 356–367 (doi: 10.1177/016173467900100406) 10.1177/016173467900100406 575833

[19] The Radio Use Website. (2015). [Online]. Available: http://www.tele.soumu.go.jp/j/ref/material/rule/ (in Japanese)

[20] Sakuma J. Anzai D. Wang J.: ‘Performance of human body communication-based wearable ECG with capacitive coupling electrodes’, Healthcare Tech. Lett., 2016, 3, (3), pp. 222–225 (doi: 10.1049/htl.2016.0023) 10.1049/htl.2016.0023 PMC5048344 27733931

[21] Noro M. Anzai D. Wang J.: ‘Common-mode noise cancellation circuit for wearable ECG’, Healthcare Tech. Lett., 2017, 4, (2), pp. 64–67 (doi: 10.1049/htl.2016.0083) 10.1049/htl.2016.0083 PMC5408556 28461900

[22] Nagai S Anzai D. Wang J.: ‘Motion artefact removals for wearable ECG using stationary wavelet transform’, Healthcare Tech. Lett., 2017, 4, (4), pp. 138–141 (doi: 10.1049/htl.2016.0100) 10.1049/htl.2016.0100 PMC5569871 28868151

